# Autonomy-supportive agents: whose support matters most, and how does it unfold in the workplace?

**DOI:** 10.1007/s12144-022-03550-9

**Published:** 2022-07-30

**Authors:** Naniki Mokgata, Leoni van der Vaart, Leon T. de Beer

**Affiliations:** 1 School of Industrial Psychology and Human Resource Management, North-West University, Potchefstroom, South Africa; 2 WorkWell Research Unit, North-West University, Potchefstroom, South Africa; 3 Optentia Research Unit, North-West University, Potchefstroom, South Africa

**Keywords:** Autonomy-supportive behaviors, Self-determination theory, Serial indirect effect, Small-medium enterprises, Well-being, Performance

## Abstract

Managers and colleagues satisfy others’ need for autonomy, but employees can also satisfy their own need by engaging in autonomy crafting practices. Although all three sources of autonomy support can benefit employee outcomes, they may not be equally beneficial. Furthermore, their benefits may not be straightforward, but rather a psychological process unfolding. To test these assumptions, the aim of the present study was twofold: to determine whether the different sources of support explained significantly different amounts of variance in autonomy satisfaction when compared and to understand the psychological process through which autonomy support from three sources influenced performance, more specifically, whether autonomy support indirectly affected performance through perceived autonomy satisfaction and work engagement in serial. In a sample of 278 employees, autonomy support from others (especially managers) and autonomy crafting played a role in autonomy satisfaction. Furthermore, the results indicated that autonomy support was associated with performance through its serial associations with autonomy satisfaction and work engagement. The results emphasized the importance of autonomy support for performance, enabling organizations to proactively design interventions to improve engagement and performance.

**Supplementary Information:**

The online version contains supplementary material available at 10.1007/s12144-022-03550-9.

The self-determination theory (SDT) suggests that employees’ need for autonomy – the need to make decisions and act in line with one’s wishes – should be satisfied (Ryan & Deci, 2017; Vansteenkiste et al., 2020), especially during times of uncertainty (Vermote et al., 2021; Laporte et al., 2022b). Globally, measures to curb the spread of the novel coronavirus threatens the livelihoods and well-being of individuals (Vermote et al., 2021) and businesses (Gregurec et al., 2021). Small and medium-sized enterprises (SMEs) have been impacted exceptionally hard by the pandemic and associated lockdown measures (Bartik et al., 2020; Kalidas et al., 2020). Coupled with an increase in the use of surveillance software by employers (Alsever, 2021), employees may feel that they have less control over their choices and behaviors (i.e., reduced satisfaction of their need for autonomy) (Jungert et al., 2020), which hampers not only their performance, but also their well-being (Sheldon et al., 2021; Vansteenkiste et al., 2020; Laporte et al., 2022b).

According to the literature, both managers and colleagues can support others’ need for autonomy (Jungert et al., 2013, 2020; Kaabomeir et al., 2022; Moreau & Mageau, 2012). More recent studies have confirmed individuals’ role in supporting their own need for autonomy as well (Laporte et al., 2021, 2022a, 2022b; Sheldon et al., 2021). Congruent with SDT assumptions, Laporte et al. (2021) introduced the concept of need crafting as a proactive side of need satisfaction. By satisfying the need for autonomy, managers, colleagues, and the self initiate a process through which employees’ performance is enhanced via enhanced well-being. This process flows from autonomy satisfaction that enhances work engagement (an indicator of employee well-being) (Coxen et al., 2021; Van den Broeck et al., 2016) and engagement that improves employee performance (Koekemoer et al., 2021; van Dorssen-Boog et al., 2021).

A few SDT studies support the positive effect of autonomy support in the work context. These studies also compared the impact of the different sources (i.e., managers versus colleagues) of autonomy support (Jungert et al., 2013, 2020; Moreau & Mageau, 2012). Although valuable, there are several unexplored areas. First, these studies only included support from the manager and colleagues, neglecting the role of the individual. This is unfortunate, as the core notion of SDT advocates for the self-determined actions of individuals. Studies that focused on self-support (sometimes in comparison to interpersonal support) included adolescents (Laporte et al., 2021, 2022a, 2022b), the general public (Laporte et al., 2022b), hikers, and students (Behzadnia & FatahModares, 2020; Sheldon et al., 2021). Job crafting and its effectiveness are influenced by the context in which employees craft (Zhang & Parker, 2019). Therefore, the value-add of need crafting in the work context should not simply be assumed. Second, mixed results were reported within and between the studies. For example, Jungert et al. (2013) reported that collegial support mattered more than managerial support over time, whereas Moreau and Mageau (2012) reported the opposite. Jungert et al. (2013) found some differences in the results of their study, with managerial support being more important for certain outcomes and collegial support for others. These mixed findings raise the question of which source matters most and whether it depends on the type of outcome measured.

Third, none of the studies included performance as an outcome. This is surprising, given its importance and the potential of need support to enhance performance (Mossman et al., 2022; Slemp et al., 2018). Fourth, none of these studies took a process approach in which the association between need-supportive behaviors and the outcomes is explained by another (i.e., mediating) variable. Mediating variables often provide valuable insights into findings, especially into the discrepancies between or within studies. Sheldon et al. (2021) alluded to this in stating that self-supportive self-talk might not have directly affected performance, but that an indirect effect might have been plausible. They also reported that need satisfaction was an essential intervening variable. Last, these studies mainly relied on hierarchical regression analysis to determine whether additional variance was explained when adding a source of support. The drawback of this technique is that it becomes challenging to isolate the effect of a single predictor when the predictors are too highly correlated (Tonidandel & LeBreton, 2015).

Therefore, the aim of the present study was twofold. First, the study sought to determine whether the different sources of autonomy support explained significantly different amounts of variance in autonomy satisfaction when compared to one another. Second, the study endeavored to understand the process through which autonomy support from three sources (i.e., manager, colleagues, and self) influenced performance, more specifically, whether autonomy support indirectly affected performance through perceived autonomy satisfaction and work engagement in serial. 

The current study contributes to the literature in three ways. First, it included (and compared) all three sources of support (using relative weight analysis that accurately partitioned the effects of correlated predictors), contributing to the debate about which source matters most. This contribution enables SDT researchers to move closer to reaching consensus. The study also included need crafting, a relatively new concept in SDT research (Laporte et al., 2021). Second, it took a process approach to the impact of need support, potentially providing valuable insights into how their impact unfolds in the workplace. Third, the study illustrated the importance of autonomy support for performance, not only for well-being. From a practical perspective, the study adds value for SMEs, as it provides suggestions for improving employee performance.

The article starts with a conceptualisation of SDT and the variables included in the current study, followed by a review of the relevant literature that support the hypotheses. Next, the paper outlines the method(ology) employed to collect and analyse the data, followed by an outline of the results. Last, it provides an interpretation of the findings, whilst contextualising it in the current body of knowledge and acknowledging the limitations.

## Literature review and hypotheses

### Self-determination theory

The SDT is deeply entrenched in the ethos of individuals’ capacity to make choices and manage their behavior instead of being controlled. In this way, SDT plays an essential role in explaining self-determined behavior in psychological health and well-being. When individuals are self-determined, they experience control over their choices and actions, which leads to internalization (of behavior) and well-being (Ryan & Deci, 2017; Ryan et al., 2021). The capacity to self-regulate is a basic psychological need within the basic psychological needs theory (BPNT). The BPNT is one of six mini-theories of SDT, and it assumes that every individual possesses three basic universal psychological needs: the need for competence, relatedness, and autonomy (Ryan & Deci, 2017). These needs are innate, and their satisfaction is universally essential for the experience of psychological growth, integration, and well-being (Ryan & Deci, 2020; Tan et al., 2021). In an occupational setting, autonomy support are associated with optimal functioning in the workplace (Slemp et al., 2018) and warrant further investigation.

### The relative importance of various sources of autonomy support for autonomy satisfaction

According to SDT, need satisfaction is a consequence of person-environment interaction, meaning that the need for autonomy is satisfied through support on an interpersonal (i.e., managers and colleagues) level (Ryan & Deci, 2017; Ryan et al., 2021; Vansteenkiste et al., 2020). Autonomy support (from others) are characterized by acknowledging an employee’s frame of reference and perspective, minimizing external incentives and threats, providing opportunities for choices and input, and avoiding controlling behaviors and language such as criticism and controlling statements (Slemp et al., 2018). Managers are in a unique position to support subordinates’ need for autonomy by taking their perspectives and providing discretion over daily work tasks, methods, and freedom of choice (Chong et al., 2021; Kaabomeir et al., 2022). Autonomy support is vital in hierarchical (Deci & Ryan, 2000) and horizontal interactions (Jungert et al., 2020). Changes in today’s world of work leave managers with a larger span of control due to the flattening of organizations, resulting in a decrease in the number of managers (Jungert et al., 2020). Therefore, colleagues have to step up to fill this void and are an essential source of autonomy support. The word ‘colleagues’ refers to individuals in the workspace with similar work experience, skills, and knowledge, and they hold the same position at a hierarchical level (Moreau & Mageau, 2012).

Autonomy support can, furthermore, occur on an intrapersonal level (i.e., the self). Congruent with the theoretical assumptions of SDT (i.e., the proactive nature of an individual), it is essential to also consider an employee’s capacity to self-create optimal conditions for basic psychological need satisfaction. This capacity is termed ‘need crafting’. Need crafting entails an awareness of need satisfaction and the ability to act toward the satisfaction of needs as a way of experiencing autonomy (Laporte et al., 2021).

Empirical studies provide support for the direct relationships between need support and its positive outcomes: motivation (Jungert et al., 2013, 2020; Kaabomeir et al., 2022), self-efficacy (Jungert et al., 2013), work satisfaction, psychological health (Moreau & Mageau, 2012), subjective well-being (Moreau & Mageau, 2012; Sheldon et al., 2021), and psychological need satisfaction (Sheldon et al., 2021). Need crafting has also been associated with need satisfaction and well-being (Laporte et al., 2021; 2022a, 2022b). Therefore, it was hypothesized that:

- Hypothesis 1a: All sources of autonomy support contributed to autonomy satisfaction.

In addition, previous studies found that the different sources of need support were not equal in their contributions to employee outcomes. For example, self-support mattered more for subjective well-being and meaning in life than authority support (Sheldon et al., 2021). On closer inspection of these studies, the results revealed that the differential contribution of each source might depend on the outcome measured. For example, managers’ support mattered more for employees’ motivation, whereas the opposite was true for self-efficacy (Jungert et al., 2013). In the presence of inconsistent evidence, a more exploratory approach is warranted. Therefore, it was hypothesized that:

- Hypothesis 1b: The explained variance in autonomy satisfaction would differ depending on the source of autonomy support.

### Autonomy support to autonomy satisfaction to work engagement to performance

Need-supportive practices are viewed as eudaemonic practices that boost need satisfaction (Martela & Sheldon, 2019). Studies support the boosting effect of such practices by reporting that need support (Kaabomeir et al., 2022; Sheldon et al., 2021) and need crafting (Laporte et al., 2021; 2022a, 2022b) are associated positively with need satisfaction. As a spin-off, need satisfaction boosts well-being (Martela & Sheldon, 2019; Laporte et al., 2022a, 2022b). This is evidenced by the positive association between autonomy satisfaction and work engagement (see Coxen et al., 2021 and Van den Broeck et al., 2016 for overviews). The internalization process may explain this spin-off, during which need satisfaction increases the likelihood of individuals engaging in activities for internal rather than external reasons (Vansteenkiste et al., 2020). As a consequence of behavioral internalization, individuals experience higher levels of well-being. Not only are they psychologically better, but they are also able to persist in their behavior (i.e., perform optimally) (Ryan & Deci, 2020; Vansteenkiste et al., 2020). Therefore, need satisfaction acts as a psychological resource that drives well-being and performance (Van den Broeck et al., 2008) and is often used as a mediator in SDT research (Ryan & Deci, 2017). For example, Laporte et al. (2022a) and (2022b) demonstrated that changes in need crafting lead to changes in need satisfaction that lead to changes in well-being. Taken together, theoretical and empirical support, for the associations between the different variables exists, together with evidence for the mediating role of need (and by implication, autonomy) satisfaction.

The current research conceptualized performance in terms of increasing uncertainty or unpredictability reflected in work role behaviors, ranging from being proficient (in meeting one’s job requirements) or adaptive (in responding to change) to being proactive (in anticipating and initiating change). These behaviors are enacted on three levels, reflecting one’s levels of contribution or independence: individual, team, and organization (Griffin et al., 2007).

Performance may not result directly from need satisfaction, but may also result from the intermediary function of work engagement. Studies have shown that work engagement leads to performance because absorbed, dedicated, and energetic employees exert more effort, which translates into higher performance (Koekemoer et al., 2021; van Dorssen-Boog et al., 2021). Furthermore, research also supported the sequential relationship between job resources, need satisfaction, work engagement, and performance (van Wingerden et al., 2018). Consequently, need satisfaction may associate positively with work engagement, which, in turn, associates positively with performance. Taken together, it was hypothesized that:

- Hypothesis 2a: Managerial autonomy support had an indirect effect on performance through perceived autonomy satisfaction and work engagement in serial, with perceived autonomy satisfaction modelled as affecting work engagement, which, in turn, affected performance.
- Hypothesis 2b: Collegial autonomy support had an indirect effect on performance through perceived autonomy satisfaction and work engagement in serial, with perceived autonomy satisfaction modelled as affecting work engagement, which, in turn, affected performance.
- Hypothesis 2c: Autonomy crafting had an indirect effect on performance through perceived autonomy satisfaction and work engagement in serial, with perceived autonomy satisfaction modelled as affecting work engagement, which, in turn, affected performance.

Only one study on autonomy support (from the self) previously included a performance measure. In their study, Sheldon et al. (2021) concluded that there was no significant relationship between autonomy support and performance. They ascribed the absence of this association to the inability of support to affect performance directly in an achievement situation. In similar vein, the main meta-analytic effect of coaches’ autonomy support on performance was small (Mossman et al., 2022). Therefore, it was expected that the direct relationship between the different sources of support and performance might be absent in the presence of the indirect effects through autonomy satisfaction and work engagement.

## Method

### Design

The current study employed a cross-sectional survey design. Although a cross-sectional design has limitations, it is suitable when relatively little is known about a topic (Spector, 2019). While some studies have investigated autonomy support from others, no published research exists for autonomy crafting or the process through which the different sources of need support exert their influence in the workplace.

### Participants

A purposive sample of employees from SMEs in South Africa participated in the study. These employees had to be between 18 and 64, employed for at least four weeks, and in possession of a Grade 12 certificate. Thirty-three participants were deleted because they failed the attention check, and seven outliers were deleted. The final sample consisted of 278 employees with a mean age of 29.30 (SD = 7.02). The average amount of time that these individuals had worked at their current organization amounted to 39.60 months (SD = 34.60), while the average amount of time that they had been reporting to their current line manager or supervisor was 30.10 months (SD = 23.50). The majority of the sample consisted of African (66.20%) females (61.20%) with at least a three-year degree or advanced diploma (61.30%). The most representative economic sector was the finances and business services sector (27.70%), followed by the community, social, and personal services sector (19.10%), and the retail, motor trade, and commercial sector (11.50%). Just over a quarter of the participants (27.30%) indicated that they only worked from home or remotely once in a while, while almost the same number of participants (24.50%) indicated that they did so most of the time.

### Measures

To obtain biographical data, participants’ level of educational qualification, age, gender, ethnicity, length of employment, duration of having reported to the manager/supervisor, the economic sector of the business, and remote working arrangements were measured. Two items (“For this question, please select option five to demonstrate your attention” and “Overall, I invested the necessary effort when answering questions in this survey”) were included in the questionnaire to evaluate the quality of data provided by participants.

The short form of the *Workplace Climate Questionnaire* (WCQ-SF; Baard et al., 2004) was used to measure employees’ perceptions of autonomy support received from others (i.e., managers and colleagues). The word ‘manager’, in this instance, referred to the most immediate supervisor. The WCQ-SF consists of six items (“I feel that my manager provides me with choices and options”) and refers to employees’ specific work setting. In measuring the autonomy-supportive behaviors of colleagues, ‘manager’ was replaced with ‘colleagues’. This framing was analogous to managerial autonomy support, except in this case, the support was received from individuals on a similar level as the individual and not from an authority figure. Consequently, the questionnaire included questions such as “I feel that my colleagues provide me choices and options”. The participants shared responses on a seven-point Likert scale, ranging from 1 (strongly disagree) to 7 (strongly agree).

The autonomy crafting subscale of the *Need Crafting Questionnaire* (NCQ; Laporte et al., 2021) was used to measure autonomy crafting. The scale explores two components of need crafting, namely, awareness and action, but only the action dimension was used for comparative purposes. Four items per need measure the action component (e.g., “I freely decide what activities I want to engage in”). The participants shared responses on a five-point Likert scale, ranging from 1 (completely not true) to 5 (completely true).

The autonomy satisfaction subscale of the *Basic Psychological Need Satisfaction and Frustration Scale – Work Domain* (BPNSFS-WD; Chen et al., 2015; Schultz et al., 2015) was used to measure the participants’ basic psychological need satisfaction. Respondents rated each of the four items by indicating the extent to which their psychological need for autonomy was satisfied (e.g., “I feel that my decisions on my job reflect what I really want”). The participants rated their responses to these questions on a seven-point Likert-type scale, ranging from 1 (strongly disagree) to 7 (strongly agree).

The ultra-short version of the *Utrecht Work Engagement Scale* (UWES-3; Schaufeli et al., 2019) was utilized to measure work engagement. The UWES structure is based on three constructs: vigor, dedication, and absorption (Schaufeli et al., 2019). Therefore, the self-report questionnaire includes three items, one for each of the subdimensions [e.g., “At my work, I feel bursting with energy” (vigor); “I am enthusiastic about my job” (dedication); and “I am immersed in my work” (absorption) (Schaufeli et al., 2019)]. A seven-point Likert scale, ranging from 0 (never) to 6 (always), was used.

The *Work Role Performance* scale (WRP; Griffin et al., 2007) was used to measure performance. The WRP scale consists of 27 items and reflects the cross-classification of tasks, teams, and organizational member behaviors with proactivity (e.g., initiates improved ways of completing tasks), adaptivity (e.g., responds constructively to changes), and proficiency (e.g., ensures that essential tasks are completed), which produces the nine subdimensions of work role performance. The self-report questionnaire uses a five-point Likert-type rating method, with response scales ranging from 1 (strongly disagree) to 5 (strongly agree).

An adaptation of the sixth version of the *Balanced Inventory of Desirable Responding* (BIDR-6; Paulhus, 1991) was used to measure self-deceptive enhancement (SDE). As one element of social desirability, SDE refers to individuals’ tendencies to portray themselves honestly, but more positively, when self-reporting (Paulhus, 1984). Evaluative measures such as performance scales are prone to biases caused by social desirability, and researchers are encouraged to measure and control social desirability when designing and conducting research (Spector et al., 2019). SDE appears to be more adequate than impression management scales to control for response bias (see Uziel, 2014). SDE was measured using five items that were selected from several studies (Asgeirsdottir et al., 2016; Hart et al., 2015; Leite & Beretvas, 2005; Pauls & Stemmler, 2003; Stöber et al., 2002) that had attempted to validate short versions of the BIDR-6. These five items had been chosen by at least four of the five validation studies for inclusion in their short forms. The items (e.g., “I always know why I like things”) are measured on a seven-point Likert-type scale, ranging from 1 (not true) to 7 (very true).

### Procedure

The relevant ethics committee approved the study (NWU-00916-21-A4). Data were collected via two avenues: social media platforms (such as LinkedIn, WhatsApp, Facebook, and Twitter) and a third-party data collection organization. A link to the QuestionPro survey was provided in the advertisement. Before completing the survey, participants were informed about key aspects of the research: the purpose of the study, grounds of confidentiality, expectations from respondents, provision of group-level feedback (optional), estimated duration of the study, publication of results, and the right to withdraw from the research without any implications. Following the information page, there was a disclaimer that participants consented to participate if they continued to complete the questionnaire. Participants from the third-party organization were rewarded for their participation, while the others stood a chance to win a shopping voucher. The processing of personal information was in line with the Protection of Personal Information Act (POPIA) 4 of 2013.

### Analytic strategy

Descriptive statistics (means, standard deviation, skewness, and kurtosis) were calculated using jamovi version 2.0.0 (The jamovi project, 2021). Applying a latent variable approach, Mplus 8.6 (Muthén & Muthén, 1998–2021) was utilized to evaluate the factor structures of the different measuring instruments [i.e., confirmatory factor analysis (CFA)]. The mean and variance-adjusted weighted least squares (WLSMV) estimator was used. The WLSMV is a robust estimator that is equipped to handle “non-normally distributed categorical data” (Wang & Wang, 2020, p. 16–17). The fit indices that were used to evaluate the fit of the measurement model (to the data) included the comparative fit index (CFI), Tucker-Lewis index (TLI), standardized root mean square residual (SRMR), root mean square error of approximation (RMSEA), chi-square (χ^2^), and degrees of freedom (*df*). A relatively good fit is indicated by CFI and TLI values exceeding 0.90. Additionally, SRMR and RMSEA values of less than 0.08 are considered indicators of good fit (Wang & Wang, 2020, pp. 20–23). Although the χ^2^ value and its significance were evaluated, doing so is “often not helpful because it is heavily influenced by sample size” (Wang & Wang, 2020, p. 20).

In the measurement model (i.e., CFA), the factor loadings of each item must be statistically significant (*p* < 0.05) to evaluate the convergent validity of the measuring instruments. Additionally, the standardized factor loadings of items should ideally exceed 0.50 (preferably 0.70), and reliability coefficients should exceed 0.70 (Hair et al., 2015). For some authors, factor loadings of 0.40 is acceptable (Netemeyer et al., 2003) and Fornell and Larcker (1981) opine that the convergent validity of a construct can still be considered adequate when the composite reliability (CR) exceeds 0.60, even if the average variance extracted (AVE) is less than 0.50. To evaluate reliability, the ordinal version of McDonald’s omega (ω) was calculated (Gadermann et al., 2012) in jamovi. Correlation coefficients were used to provide information on the size and direction of the relationships between variables. The cut-off values for the effect sizes for correlations in the study were interpreted as follows: *r* ≥ 0.10 (small effect), and *r* ≥ 0.30 (medium effect) to *r* ≥ 0.50 (large effect) (Cohen, 1992).

To establish the relative importance of the different sources of autonomy support for autonomy need satisfaction, relative weights analysis (RWA) was implemented. This procedure assists in estimating the proportion of the total variance reflected in an outcome variable that is attributable to each of the predictors. In addition, the procedure indicates whether there are significant differences in the contributions of the predictors (LeBreton & Tonidandel, 2008; Tonidandel & LeBreton, 2015). For RWA, the guidelines and web-based application (RWA-Web) created by Tonidandel and LeBreton (2015) were used. This implementation of RWA also tests statistical significance by generating bias-corrected confidence intervals (CIs) at the 95% level (95% CIs) for the calculated weights, using 10 000 bootstrapped resampling replications of the data. The results (i.e., variance expressed as a percentage) obtained from RWA can be interpreted as using effect size guidelines: *R*^2^ =  ≥ 0.01 (small effect) and *R*^2^ =  ≥ 0.09 (medium effect) to *R*^2^ =  ≥ 0.25 (large effect) (Cohen, 1992).

Mediation analysis was conducted to evaluate the specific and serial indirect effects of autonomy satisfaction and work engagement. The procedure prescribed by Hayes (2022) was used, consisting of bootstrapping with a minimum of 5 000 samples. More specifically, the PROCESS macro for R (Hayes, 2022) with model 6 was used in RStudio (R Core Team, 2021). Using bootstrapping, 95% CIs were generated, and if the upper and lower bounds of the intervals did not include zero, a meaningful indirect effect was considered present (Hayes, 2022). Given the critique associated with indirect effects testing in cross-sectional studies, Hayes (2022) recommends that researchers should control for confounding or epiphenomenal associations (i.e., controlling for variables – other than those hypothesized – that may influence the mediator[s] and ‘outcome’ variables). For this reason, the other two sources of support were included as covariates to control their effect in each mediation model. SDE, tenure, length of reporting to manager, and remote work were also included as covariates. To further account for potential confounding or spurious associations, the interaction between the three sources of support (X) and the two mediators (M; autonomy satisfaction and work engagement) was tested in the mediation analysis (as discussed by Hayes, 2022) by specifying xmtest = 1 in the PROCESS command line. For each source of support (X), there are three tests: one for each mediator in the performance model and one for autonomy satisfaction (M_1_) in the work engagement (M_2_) model. First, the interaction between the source of support and any mediator is constrained to zero, after which this constraint is released for each path leading from a mediator to another variable. All other effects of the other mediator are assumed to be independent of the source of support. An *F*-ratio is provided, comparing the constrained with the unconstrained model (Hayes, 2022).

## Results

A measurement model was specified with six correlated factors: managerial autonomy support (with six indicators), collegial autonomy support (with six indicators), autonomy crafting (with four indicators), autonomy satisfaction (with four indicators), work engagement (with three indicators), and performance (higher-order factor with nine first-order factors, each with three indicators). Although the model fitted the data well (SB-χ^2^ = 2121.73, *p* < 0.001; *df* = 1151; CFI = 0.92; TLI = 0.92; RMSEA = 0.055 [0.051, 0.059], *p* = 0.012; SRMR = 0.08), the AVE of 0.29 and the composite reliability of 0.57 (of autonomy crafting) were lower than the recommended values of 0.50 and 0.70, respectively. Reliability analysis (specifically the polychoric correlation matrix) indicated that the negatively phrased item (“Sometimes, I seem to forget to do the things that I really want to do”) correlated poorly with the other items (*r* = -0.13). Consequently, this item was removed. The revised model also fitted the data well: SB-χ^2^ = 2094.57, *p* < 0.001; *df* = 1103; CFI = 0.91; TLI = 0.91; RMSEA = 0.058 [0.054, 0.062], *p* < 0.001; SRMR = 0.08). The factor loadings, average variance extracted, and CRs of the revised model are reported in Table S1 (see supplementary file). All the factor loadings were statistically significant. They ranged as follows for the different factors: managerial autonomy support (0.71–0.88), collegial autonomy support (0.73–0.89), autonomy crafting (0.30–0.90), autonomy satisfaction (0.73–0.85), engagement (0.56–0.92), and performance (0.52–0.84). Although the AVE (0.40) and CR (0.63) of autonomy crafting was still slightly below the recommended values, the CR exceeded 0.60 and is considered acceptable for convergent validity even with an AVE of less than 0.50 (Fornell & Larcker, 1981).

Descriptive statistics (i.e., means and standard deviations) and reliability and correlation coefficients are reported in Table 1. The *p*-values of the Shapiro–Wilk test were significant for all the latent variables in this model. Most of the measuring instruments had acceptable reliability (ranging from 0.83 to 0.95). The only exception was the reliability coefficient of autonomy crafting, with a value of 0.65. To our knowledge, the autonomy crafting scale has not been used in the work context, classifying its use in the current study as exploratory. For exploratory research, a coefficient of 0.60 and higher is acceptable (Nunnally & Bernstein, 1994).

**Table 1:** Means, standard deviations, and reliability and correlation coefficients

	Mean	SD	*α*	*ω*	MAS	CAS	AC	AUTSAT	ENGAGE
1. MAS	5.06	1.29	.92	.94	-				
2. CAS	5.25	1.1	.91	.95	0.22^***^	-			
3. AC	3.49	0.75	.62	.65	0.32^***^	0.14^*^	-		
4. AUTSAT	4.63	1.33	.84	.90	0.66^***^	0.32^***^	0.44^***^	-	
5. ENGAGE	5.10	1.11	.81	.83	0.42^***^	0.20^***^	0.26^***^	0.69^***^	-
6. PERFORM	n/a	n/a	.93	.94	0.25^***^	0.24^***^	0.20^**^	0.41^**^	0.45^***^

*Notes*. SD = standard deviation; α = ordinal Cronbach’s alpha (reported for transparency purposes); *ω* = ordinal McDonald’s omega; MAS = managerial autonomy support; CAS = collegial autonomy support; AC = autonomy crafting; AUTSAT = autonomy satisfaction; ENGAGE = work engagement; PERFORM = higher-order latent factor of performance components; n/a = not applicable. *p .05, **p < .01, ***p < .001

The associations between the variables were all significant and in the expected direction (i.e., positive), supporting the convergent validity of the instruments. In general, the associations between the different types of autonomy support ranged from small (*r* = 0.14) to medium (*r* = 0.32). The associations between the sources of support and autonomy satisfaction ranged from medium (*r* = 0.32) to large (*r* = 0.66), whereas their associations with work engagement ranged from small (*r* = 0.20) to medium (*r* = 0.42). Their associations with performance were small (*r* = 0.20–0.25), an early indication that they influenced performance indirectly rather than directly. The association between autonomy satisfaction and work engagement was large (*r* = 0.69), whereas its association with performance was medium (*r* = 0.41). The association of work engagement with performance was similar, in that it was also medium (*r* = 0.45). The associations between the antecedents and the different performance variables are reported in Table S2 (see supplementary file).

Factor scores were exported from the measurement model and used in the RWA and mediation analysis. Results from the RWA-Web software are shown in Tables 2 and 3.

**Table 2:** Relative weight analysis results with autonomy satisfaction as the criterion variable

Criterion = autonomy satisfaction (*R*^2^ = 61.88%)			
	Raw relative weight	95% CI	Rescaled relative weight (%)
Managerial autonomy support	.39	[.32, .46]	62.88
Collegial autonomy support	.08	[.03, .13]	12.35
Autonomy crafting	.15	[.09, .22]	24.77

*Notes.* CI = confidence interval

**Table 3:** Comparing the contributions of the different sources of autonomy support

	Lower 95% CI	Upper 95% CI	Result
**Reference determinant: managerial autonomy support**			
Collegial autonomy support	-.40	-.22	Meaningfully lower
Autonomy crafting	-.34	-.13	Meaningfully lower
**Reference determinant: collegial autonomy support**			
Autonomy crafting	-.01	.16	No meaningful difference
Managerial autonomy support	.22	.41	Meaningfully higher
**Reference determinant: autonomy crafting**			
Managerial autonomy support	.13	.34	Meaningfully higher
Collegial autonomy support	-.16	.00	No meaningful difference

*Notes.* CI = confidence interval

The 95% CIs for the three determinants indicated their significance: managerial [0.32, 0.46], collegial [0.03, 0.13], and crafting [0.09, 0.22]. Therefore, support existed for Hypothesis 1a. Table 2 includes the antecedent labels, their raw relative weights with bias-corrected 95% bootstrapped CIs, and the rescaled relative weight expressed as the percentage of variance explained in autonomy satisfaction. As shown in Table 2, all the explained variances were meaningful (the CIs did not include zero), and the model could explain 61.88% of the total variance in autonomy satisfaction. Using cut-off values for effect sizes, this is considered a large effect. More specifically, the rescaled relative weights showed that managerial autonomy support (62.88%) explained more than half of the variance, followed by autonomy crafting (24.77%), with collegial autonomy support (12.35%) explaining the least amount of variance in autonomy satisfaction.

When comparing the different sources of autonomy support for RWA (see Table 3), managerial autonomy support explained significantly more variance in autonomy satisfaction than both collegial autonomy support and autonomy crafting but the latter two did not differ statistically significantly from each other. Therefore, some support existed for Hypothesis 1b.

Before conducting the serial mediation analyses, a measurement model for the SDE variable was specified. The model fitted the data well: (SB-χ^2^ = 18.73, *p* < 0.001; *df* = 5; CFI = 0.96; TLI = 0.93; RMSEA = 0.101 [0.055, 0.152], *p* = 0.036; SRMR = 0.03). RMSEA values can be artificially high for models with low degrees of freedom and indicates poor model fit even though the other fit indices indicate the opposite (Kenny et al., 2015). Although some authors argue that this fit statistic should not be calculated when the degrees of freedom is low, we decided to report this statistic for transparency purposes. Recent simulation studies have also shown that the SRMR performs better than the RMSEA with ordered categorical indicators (Shi et al., 2020). The SRMR value showed a good fit between the model and the data. Considering also the other fit statistics, we can conclude acceptable model fit. Further supporting this decision, is the fact that most factor loadings were acceptable (ranging between λ = 0.51 and 0.78), except for the reversed item (Item 3), which had a loading of λ = -0.38. Nonetheless, the loading was still statistically significant (*p* < 0.001). The reliability coefficient for the scale was also acceptable (*ω* = 0.73), providing further evidence for its convergent validity. The average score for SDE was 4.82 (SD = 0.99). The *p*-values of the Shapiro–Wilk test were non-significant, indicating that there was support for the null hypothesis that the data were normally distributed. The factor scores for SDE were exported and used as covariates in the mediation analysis. SDE (as covariate of the mediation analyses) was not significantly associated with autonomy satisfaction (β = 0.14, *p* = 0.07) in any of the three models reported below. However, it was significantly associated with work engagement (β = 0.16, *p* = 0.03) and performance (β = 0.16, *p* = 0.001) in all three of the models. We also included tenure, length of reporting to manager, and frequency of remote work as control variables with all of them being unrelated to autonomy satisfaction (β = -0.00, *p* = 0.44; β = -0.00, *p* = 0.59; β = 0.02, *p* = 0.29) and work engagement (β = 0.00, *p* = 0.31; β = -0.00, *p* = 0.22; β = -0.04, *p* = 0.06). Tenure (β = 0.00, *p* = 0.79) and length of reporting to manager (β = -0.00, *p* = 0.86) were also unrelated to performance but remote work (β = -0.04, *p* < 0.01) was significantly (yet negatively) related to performance.

Three separate serial indirect effect models were specified for the determinants (i.e., three sources of support). Each of the three models had two mediators, namely, autonomy satisfaction and work engagement, and the source of the autonomy support (i.e., manager, colleague, and self) was specified as having its indirect effect on performance via the two mediators. This specification resulted in three indirect effects (that added up to the total indirect effect): one through autonomy satisfaction, one through work engagement, and one through autonomy satisfaction and work engagement (in serial). The remaining effect was the direct association between the source of support and performance. In each of these models, the other two sources of support were also included as covariates to control for their effect. The unstandardized results of these serial models are reported in Table 4.

**Table 4:** Serial indirect effect of managerial and collegial autonomy support and autonomy crafting on performance through autonomy satisfaction and engagement

Indirect effect	Estimate	SE	95% CI
***Managerial autonomy support***			
Total indirect effect	.11	.03	[.06, .16]
MAS → AUTSAT → PERFORM	.05	.03	[-.00, .11]
MAS → ENGAGE → PERFORM	-.02	.01	[-.04, -.00]
MAS → AUTSAT → ENGAGE → PERFORM	.07	.02	[.03, .11]
***Collegial autonomy support***			
Total indirect effect	.03	.01	[.00, .06]
CAS → AUTSAT → PERFORM	.02	.01	[-.00, .04]
CAS → ENGAGE → PERFORM	-.01	.01	[-.03, .00]
CAS → AUTSAT → ENGAGE → PERFORM	.02	.01	[.01, .04]
***Autonomy crafting***			
Total indirect effect	.12	.05	[.03, .22]
AC → AUTSAT → PERFORM	.07	.04	[-.00, .15]
AC → ENGAGE → PERFORM	-.03	.02	[-.07, .01]
AC → AUTSAT → ENGAGE → PERFORM	.09	.03	[.03, .16]

*Notes.* SE = standard error; CI = confidence interval; MAS = managerial autonomy support; CAS = collegial autonomy support; AC = autonomy crafting; AUTSAT = autonomy satisfaction; ENGAGE = work engagement; PERFORM = performance

From Table 4, it is evident that the data were consistent with the claim that managerial autonomy support had an indirect effect on performance, serially, through autonomy satisfaction and work engagement and through work engagement independent of autonomy satisfaction. However, the data were not consistent with the claim that managerial autonomy support associated with performance through autonomy satisfaction independent of work engagement. Two indirect effects were significant in this case; therefore, we compared whether the two differed from each other. For this purpose, we used the contrast option in PROCESS. In the comparison equation, the second indirect effect (through engagement) was subtracted from the third indirect effect (i.e., the serial indirect effect) and provided an estimated value of -0.19. The results indicated that the 95% CIs [-0.31, -0.08] comparing these indirect effects did not contain zero, meaning that the two effects differed significantly and that the serial indirect effect was larger than the single indirect effect. Therefore, support existed for Hypothesis 2a.

**Fig. 1:**
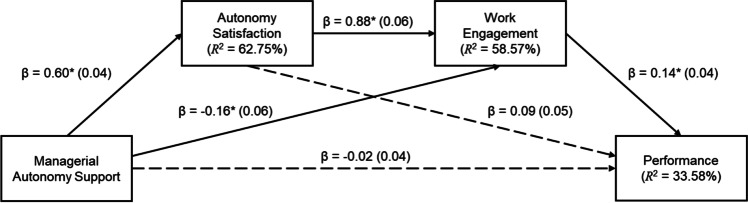
Serial indirect effect of managerial autonomy support on performance through autonomy satisfaction and engagement. *Notes.* Standard errors are reported in brackets; dashed lines indicate non-significant associations; ^*^ p < .01; for simplification, the covariates (CAS, AC, SDE, tenure, length of reporting to manager, and remote work) were omitted from the figure

**Fig. 2:**
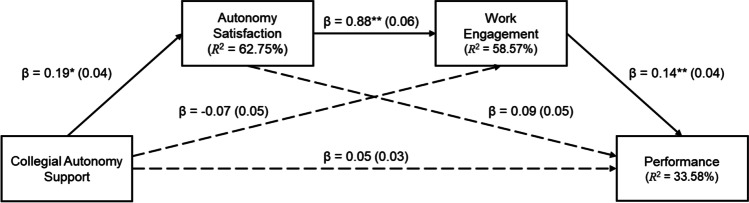
Serial indirect effect of collegial autonomy support on performance through autonomy satisfaction and engagement. *Notes.* Standard errors are reported in brackets; dashed lines indicate non-significant associations; ^*^ p < .01; for simplification, the covariates (MAS, AC, SDE, tenure, length of reporting to manager, and remote work) were omitted from the figure

**Fig. 3:**
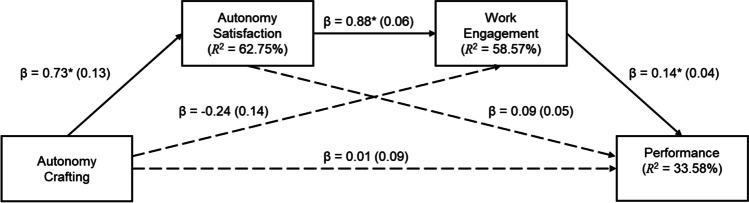
Serial indirect effect of autonomy crafting on performance through autonomy satisfaction and engagement. *Notes.* Standard errors are reported in brackets; dashed lines indicate non-significant associations; ^*^ p < .01; for simplification, the covariates (MAS, CAS, SDE, tenure, length of reporting to manager, and remote work) were omitted from the figure

Figure 1 shows that there was no direct association between managerial autonomy support and performance. In addition to the relationships depicted in this figure, collegial autonomy support (as a covariate) was significantly associated with autonomy satisfaction, but not with engagement or performance. Autonomy crafting (as a covariate) was significantly associated with autonomy satisfaction, but not with work engagement or performance. The values for the associations are the same as those reported in Figs. 2 and 3 and are, therefore, not repeated here.

From Table 4, it is evident that the data were consistent with the claim that collegial autonomy support had an indirect effect on performance, serially, through autonomy satisfaction and work engagement, but not through work engagement independent of autonomy satisfaction or through autonomy satisfaction independent of work engagement. Therefore, support existed for Hypothesis 2b.

Figure 2 shows that there was no direct association between collegial autonomy support and performance. In addition to the relationships depicted in this figure, managerial autonomy support (as a covariate) was significantly associated with autonomy satisfaction and work engagement, but not with performance. Autonomy crafting (as a covariate) was significantly associated with autonomy satisfaction, but not with work engagement or performance. The values for the associations are the same as those reported in Figs. 1 and 3 and are, therefore, not repeated here.

From Table 4, it is evident that the data were consistent with the claim that autonomy crafting had an indirect effect on performance, serially, through autonomy satisfaction and work engagement, but not through work engagement independent of autonomy satisfaction or through autonomy satisfaction independent of work engagement. Therefore, support existed for Hypothesis 2c.

Figure 3 shows that there was no direct association between autonomy crafting and performance. In addition to the relationships depicted in this figure, managerial autonomy support (as a covariate) was significantly associated with autonomy satisfaction and work engagement, but not with performance. Collegial autonomy support (as a covariate) was significantly associated with autonomy satisfaction, but not with work engagement or performance. The values for the associations are the same as those reported in Figs. 1 and 2 and are, therefore, not repeated here.

Results of the tests for the interaction between the autonomy support variables (X) and each mediator (M) are presented in Table 5. These results support the assumptions of no meaningful interactions (*p* ≤ 0.05) between the autonomy support variables (X) and either mediator in this serial indirect effect model, obviating confounding associations.

**Table 5:** Tests of autonomy support by autonomy satisfaction and work engagement

Outcome	Interaction (M*X)	*F*	d*f*1	d*f*2	*p*
***Managerial autonomy support (MAS)***					
Work engagement	AUTSAT*MAS	.02	1.00	259.00	.90
Performance	AUTSAT*MAS	.08	1.00	258.00	.78
Performance	ENGAGE*MAS	1.20	1.00	258.00	.28
***Collegial autonomy support (CAS)***					
Work engagement	AUTSAT*CAS	.03	1.00	259.00	.87
Performance	AUTSAT*CAS	.03	1.00	258.00	.87
Performance	ENGAGE*CAS	.91	1.00	258.00	.34
***Autonomy crafting (AC)***					
Work engagement	AUTSAT*AC	.37	1.00	259.00	.54
Performance	AUTSAT*AC	.02	1.00	258.00	.90
Performance	ENGAGE*AC	.10	1.00	258.00	.75

*Notes. F* = *F*-ratio; d*f* = degrees of freedom; *MAS* = managerial autonomy support; CAS = collegial autonomy support; AC = autonomy crafting; AUTSAT = autonomy satisfaction; ENGAGE = work engagement; PERFORM = performance

## Discussion

The aim of the present study was twofold: to determine whether the different sources of support explained significantly different amounts of variance in autonomy satisfaction and whether autonomy support from three different sources (i.e., manager, colleagues, and self) had an indirect impact on employees’ work performance through autonomy satisfaction and work engagement, in serial mediation.

In line with SDT, interpersonal and intrapersonal support contributes to autonomy satisfaction (Ryan & Deci, 2017). Expanding on the propositions of the SDT, the results of this study provided some support for the hypothesis that the three sources did not contribute equally to autonomy satisfaction. Employees reporting to managers who understood and offered choices and options were likely to experience a sense of freedom and alignment between their actions and interests (or wishes). The same went for employees who received these autonomy support from their colleagues, but to a lesser extent than when managers showed these behaviors. Similar findings were reported in previous studies; for example, managerial support mattered more for motivation (Jungert et al., 2013), intention to leave, and psychological distress (Moreau & Mageau, 2012) than collegial support. The findings also revealed that employees actively engaging in activities aligned with their interests and wishes (i.e., crafting for autonomy) contributed to a sense of freedom, but not meaningfully more than passively receiving such autonomy support from colleagues. However, crafting still played ‘second fiddle’ to managerial support, which contradicted previous research (Sheldon et al., 2021), in which crafting mattered more than support from an authority figure.

There are three plausible explanations for this contradiction: context, the type of need measured, and virtual work arrangements. First, the literature revealed that context played a crucial role in employees’ perceptions when evaluating the contribution of the different sources of autonomy support (Jungert et al., 2020). The present study was conducted in the small business work context, which may present different dynamics when compared to an educational context (with large groups of introductory psychology students) as was the context for Sheldon et al.’s (2021) research. In the work context, managers fulfil four roles: planning, organizing, leading, and controlling (Fayol, 1916, as cited in Robbins & Judge, 2019). In a small business context, managers (and often the owners) deal with these operational and employee-related matters directly, daily (Nyatyowa, 2017). Consequently, they are frequently and hands-on involved in matters where employees may be provided with choices (or not). Although the role of a lecturer overlaps with that of a manager in many ways, interactions (in large public institutions) are less frequent, more distant, and most likely do not result in decisions that affect livelihoods. Hence, students may have more opportunities to craft their autonomy and feel more psychologically safe to do so, as it will not necessarily determine (immediate) employment and income outcomes.

Second, it is also plausible that the type of need support played a role. Managers may have a more significant impact on autonomy satisfaction than, for example, on relatedness satisfaction due to the more transactional nature of traditional hierarchical interactions. Third, more than half of the sample indicated they worked from home at least half the time. Although this may provide them with more opportunities to craft, managers may react to this new way of working by micromanaging, which frustrates the need for autonomy. This micromanagement is evident in the increase in subscriptions to ‘spy software’ that enables managers to keep track of employees’ activities on their computers (Mosendz & Melin, 2020). As this was the first study of its kind (i.e., comparing three sources of autonomy support in the work context), more research is needed to test these tentative explanations.

The results of this study also supported the eudaemonic nature of need-supportive practices and their cascading nature (Martela & Sheldon, 2019). As hypothesized, autonomy support was associated indirectly with performance through its serial indirect (cascading) effect. SME employees performed their tasks more optimally while also preparing for, and adapting to, change (on all three levels) if their managers and colleagues created social contexts that provided them with options to make decisions and also when they had the freedom to design their tasks in a way that was aligned with their interests and wishes. They performed more optimally because they experienced more autonomy, which, in turn, made them more energetic, absorbed in, and dedicated to, their tasks. The findings were in line with recent studies that support the (direct) positive association between autonomy satisfaction and need support (Kaabomeir et al., 2022; Sheldon et al., 2021) and need crafting (Laporte et al., 2021, 2022a, 2022b), and the (direct) positive association between autonomy satisfaction, engagement, and performance (Coxen et al., 2021; Van den Broeck et al., 2016). Previous studies also supported the (direct) positive association between engagement and performance (Koekemoer et al., 2021; van Dorssen-Boog et al., 2021; van Wingerden et al., 2018). The findings are also aligned with previous studies that supported the mediating role of need satisfaction (Laporte et al., 2022a, 2022b) and the sequential effect of resources on performance (van Wingerden et al., 2018). However, it is worth mentioning that the direct associations between the different sources of support and outcomes (in this case, work engagement and performance) were not significant. This contradicts previous organizational research that reported significant direct associations between need support and outcomes like learning (Liu & Fu, 2011), motivation (Jungert et al., 2013, 2020; Kaabomeir et al., 2022), self-efficacy (Jungert et al., 2013), work satisfaction, psychological health (Moreau & Mageau, 2012), and subjective well-being (Moreau & Mageau, 2012). However, it supports our theorizing that need support initiates a process through which it exerts its effects and would coincide with Sheldon et al.’s (2021) assertion that the need support-performance link is indirect rather than direct. Also, if meta-analytic findings (Mossman et al., 2022) show that autonomy support has a small main effect on performance, it is expected that this variance may decrease as mediators are introduced to the process.

The present study enhances our understanding of autonomy support in the work context in several ways. First, the study illustrated that autonomy support from others and autonomy crafting played a role in autonomy satisfaction. To date, SDT research has focused more on the role of the interpersonal context in need-based experiences and neglected the role of the self. Second, the results of the study supported the benefits of autonomy crafting in the work context, similar to the benefits reported among adolescents and the general public (see Laporte et al., 2021; 2022a, 2022b). Thus, early indications are that need crafting transcends context. Third, the benefits reported did not exceed those derived from collegial support, but could not trump managerial support. Similar to a previous study that included a motivational construct in evaluating the differential impact of two sources of support (i.e., managerial and collegial) (Jungert et al., 2013), the present study also concluded that managerial support was more important than collegial support. Altogether, these findings might hint at an early conclusion (in the comparison of sources debate) that support, especially autonomy support from managers, might be more important (at least for motivation) than such behaviors from colleagues (or the self). Last, evidence was provided for why need support (and specifically autonomy support) might not directly affect performance. Instead, need support initiated a psychological process that enhanced need-based experiences. Need-based experiences (or satisfaction) as a psychological resource enhanced work-related well-being (i.e., work engagement), facilitating the internalization and persistence of behavior.

## Implications for practice

Small and medium enterprise (SME) employers can improve employee performance by leveraging the motivational frameworks of the self-determination theory (SDT) and work engagement. From an SDT perspective, employers can focus on the antecedents of autonomy support and autonomy crafting behaviors. From a work engagement perspective, the job demands-resources (JD-R) theory (Bakker & Demerouti, 2017) would be helpful.

Matosic and colleagues identified several antecedents of need-supportive communication style, which they classified into contextual factors (e.g., social support, pressure from authorities), perceptions of others’ behaviors and motivations (e.g., perceptions of others’ self-determination), and personal factors (e.g., employees’ own autonomy satisfaction; Matosic et al., 2016). Organizational interventions can, thus, focus on creating contexts that enable employees (especially managers) to be autonomy-supportive. Such contexts do not place unnecessary pressure on managers to maximize subordinates’ performance through controlling statements and encouraging teamwork for social support. Research suggests that people can be trained to become more autonomy-supportive in their interactions (Jungert et al., 2020), and therefore, leadership development interventions may be beneficial. Such interventions can focus on leaders seeking opportunities to satisfy their own needs for autonomy and being informed of what constitutes autonomy-supportive behaviors (Rocchi et al., 2017).

Another promising avenue is to focus on motivational work designs that stimulate the need for autonomy. More specifically, an agentic work design is recommended, where employees have greater autonomy, control, and influence over their work tasks, activities, relationships, and responsibilities. This work design will simultaneously promote engagement and performance (Gagné et al., 2021). Apart from autonomy, other job resources such as providing meaningful performance feedback and social support can also enhance work engagement and performance (Bakker & Demerouti, 2017).

Need crafting training interventions are recommended as well. Borrowing from the literature on job crafting interventions (Demerouti et al., 2021; Hulshof et al., 2020; van Wingerden et al., 2017), a need crafting intervention can focus on teaching employees about autonomy crafting and its benefits, allowing them to analyze their job to identify opportunities to craft their autonomy, developing a crafting plan, and identifying the obstacles and resources needed when implementing their plan. Crafting does not happen in isolation; therefore, organizational climates and job designs should be supportive. From a climate perspective, an open, proactive, and supportive climate would be helpful. From a design perspective, employees should have the latitude to craft their jobs (Lazazzara et al., 2020).

## Limitations and recommendations for future research

The contribution of the current study should be considered in light of several methodological and theoretical limitations. First, the study’s design was correlational, and although the hypotheses of the study were based on theoretical propositions from SDT and the JD-R theory, causal inferences could not be made. By definition, cross-sectional designs involve collecting data from a participant only once, and definite conclusions about the direction of the relationships cannot be drawn (Spector, 2019). Future research should use longitudinal or experimental designs to understand the causal influence of autonomy support and crafting on performance (via autonomy satisfaction and work engagement). Covariates (e.g., tenure, length of reporting to manager, and remote work) were also included to control for their associations with autonomy satisfaction, work engagement, and performance. Despite the indirect effect being present whilst holding these covariates constant, we cannot exclude the possibility of other confounding or epiphenomenal associations with variables not measured in the current study. For this reason, we recommend that future experimental studies include other theoretically plausible covariates.

Second, the data were obtained from one source: the employee. If two or more measures use the same method, the strength of their associations may be inflated (Spector et al., 2019). Self-report performance measures are also critiqued for their potential to create biased data (Koopmans et al., 2014; Widyastuti & Hidayat, 2018). Fortunately, statistical research has shown that multivariate analyses, such as those reported in the present study, are unlikely to result in inflated estimates of relations as these analyses are naturally controlled for shared method variance.^1^ Although employees are often the best source of information (regarding their psychological state), and others’ performance reports may suffer from the same bias as self-report, more objective measures (especially for performance) may be recommended. In the current study, we aimed to control for the bias introduced by self-report (of performance) by measuring SDE as recommended by Spector et al. (2019). Still, data from different sources may be superior.

Third, generalizability was a limitation. Given that the sample consisted of SME employees, generalizing the results to the larger workforce is discouraged. Random probability sampling methods were not employed in the absence of a list of SME employees in South Africa. Future research should gather data using a random sample from SMEs in South Africa and elsewhere. Measuring the size of the organization could also be meaningful in explaining some of the findings from studies such as the present. Last, the need crafting scale of Laporte et al. (2021) was designed to be generic. Therefore, its items might not have mapped the full range of behaviors an employee would display in the work context. The generic nature of the scale (and possibly the use of one part of a subscale) could also have influenced its psychometrics properties. It is also worth mentioning that the scale was developed for Flemish speaking individuals and may not perform the same in a non-Western, multilingual context. Therefore, researchers are advised to develop a work-specific scale and investigate its associated measurement invariance for future application.

From a theoretical perspective, the present study was limited in its focus on the positive side of SDT, overlooking the dark side of frustrating employees’ needs for autonomy (through autonomy need-thwarting behaviors) and its associated outcomes. Given that need-thwarting behaviors and need frustration are not merely the opposite of need-supportive behaviors and need satisfaction (Rocchi et al., 2017; Vansteenkiste et al., 2020), future research should consider the inclusion of the dark side of human functioning (Coxen et al., 2021; Van den Broeck et al., 2016). The present study only included one of the three basic psychological needs (for reasons mentioned earlier), and future studies could consider the inclusion of competence and relatedness. In line with more recent work in the field (Laporte et al., 2022a, 2022b), it is also recommended that organizational researchers evaluate the role of managerial and collegial autonomy support in predicting autonomy crafting of employees and/or their moderating the effect.

## Supplementary Information

Below is the link to the electronic supplementary material.

Supplementary file1 (DOCX 31 KB)

## Data Availability

The datasets generated during and/or analysed during the current study are available from the corresponding author on reasonable request.

## Footnotes

1 We employed Harman’s single-factor test to address the issue of common method variance (Podsakoff et al., 2003). Results showed that the single factor only accounts for 18.64% of the variance which is far below the cut-off value of 50% that is deemed problematic.
