# Supplementary material for: Autonomy-supportive agents: whose support matters most, and how does it unfold in the workplace?
Source: Curr Psychol. 2022 Jul 30:1–16. Online ahead of print. doi: 10.1007/s12144-022-03550-9 (PMC9362695; doi:10.1007/s12144-022-03550-9)
Supplement: Supplementary file 1 — Supplementary file1 (DOCX 31 KB) [file 12144_2022_3550_MOESM1_ESM.docx]

**Supplementary file**

**Table S1**

*Standardised Factor Loadings (λ), Standard Errors, Average Variance Extracted, and Critical Ratios for the Final Measurement Model (n = 278)*

| **Items** | ***λ*** | **S.E.** | ***p*-value** | **AVE** | **C.R.** |
| --- | --- | --- | --- | --- | --- |
| **1. Managerial autonomy support** | | | | 0.66 | 0.92 |
| MAS1 | 0.80 | 0.03 | 0.001 |  |  |
| MAS2 | 0.84 | 0.02 | 0.001 |  |  |
| MAS3 | 0.80 | 0.03 | 0.001 |  |  |
| MAS4 | 0.71 | 0.03 | 0.001 |  |  |
| MAS5 | 0.88 | 0.02 | 0.001 |  |  |
| MAS6 | 0.82 | 0.02 | 0.001 |  |  |
| MAS1 | 0.80 | 0.03 | 0.001 |  |  |
| **2. Collegial autonomy support** | | | | 0.65 | 0.92 |
| CAS1 | 0.77 | 0.03 | 0.001 |  |  |
| CAS2 | 0.82 | 0.03 | 0.001 |  |  |
| CAS3 | 0.80 | 0.03 | 0.001 |  |  |
| CAS4 | 0.73 | 0.03 | 0.001 |  |  |
| CAS5 | 0.89 | 0.02 | 0.001 |  |  |
| CAS6 | 0.82 | 0.03 | 0.001 |  |  |
| **3. Autonomy crafting** | | | | 0.40 | 0.63 |
| ACT6 | 0.30 | 0.09 | 0.001 |  |  |
| ACT7 | 0.90 | 0.10 | 0.001 |  |  |
| ACT8 | 0.55 | 0.07 | 0.001 |  |  |
| **4. Autonomy satisfaction** | | | | 0.61 | 0.86 |
| AUS1 | 0.85 | 0.03 | 0.001 |  |  |
| AUS2 | 0.78 | 0.03 | 0.001 |  |  |
| AUS3 | 0.78 | 0.03 | 0.001 |  |  |
| AUS4 | 0.73 | 0.04 | 0.001 |  |  |
| **5. Work engagement** | | | | 0.62 | 0.82 |
| ENG1 | 0.83 | 0.03 | 0.001 |  |  |
| ENG2 | 0.92 | 0.03 | 0.001 |  |  |
| ENG3 | 0.56 | 0.05 | 0.001 |  |  |
| **6. Individual task performance** | | | | 0.60 | 0.82 |
| ITP1 | 0.78 | 0.06 | 0.001 |  |  |
| ITP2 | 0.76 | 0.06 | 0.001 |  |  |
| ITP3 | 0.80 | 0.06 | 0.001 |  |  |
| **7. Individual task adaptivity** | | | | 0.52 | 0.76 |
| ITA1 | 0.64 | 0.05 | 0.001 |  |  |
| ITA2 | 0.70 | 0.04 | 0.001 |  |  |
| ITA3 | 0.81 | 0.05 | 0.001 |  |  |

**Table S1 (*continues…*)**

| **Items** | ***λ*** | **S.E.** | ***p*-value** | **AVE** | **C.R.** |
| --- | --- | --- | --- | --- | --- |
| **8. Individual task proactivity** | | | | 0.75 | 0.90 |
| ITPA1 | 0.85 | 0.03 | 0.001 |  |  |
| ITPA2 | 0.90 | 0.02 | 0.001 |  |  |
| ITPA3 | 0.85 | 0.03 | 0.001 |  |  |
| **9. Team member proficiency** | | | | 0.53 | 0.77 |
| TP1 | 0.71 | 0.06 | 0.001 |  |  |
| TP2 | 0.89 | 0.06 | 0.001 |  |  |
| TP3 | 0.55 | 0.07 | 0.001 |  |  |
| **10. Team member adaptivity** | | | | 0.53 | 0.77 |
| TA1 | 0.65 | 0.04 | 0.001 |  |  |
| TA2 | 0.81 | 0.03 | 0.001 |  |  |
| TA3 | 0.71 | 0.04 | 0.001 |  |  |
| **11. Team member proactivity** | | | | 0.77 | 0.91 |
| TPA1 | 0.84 | 0.02 | 0.001 |  |  |
| TPA2 | 0.92 | 0.02 | 0.001 |  |  |
| TPA3 | 0.88 | 0.02 | 0.001 |  |  |
| **12. Organizational member proficiency** | | | | 0.59 | 0.81 |
| OP1 | 0.64 | 0.04 | 0.001 |  |  |
| OP2 | 0.77 | 0.03 | 0.001 |  |  |
| OP3 | 0.89 | 0.03 | 0.001 |  |  |
| **13. Organizational member adaptivity** | | | | 0.58 | 0.80 |
| OA1 | 0.71 | 0.04 | 0.001 |  |  |
| OA2 | 0.74 | 0.04 | 0.001 |  |  |
| OA3 | 0.82 | 0.04 | 0.001 |  |  |
| **14. Organizational member proactivity** | | | | 0.72 | 0.88 |
| OPA1 | 0.79 | 0.03 | 0.001 |  |  |
| OPA2 | 0.84 | 0.03 | 0.001 |  |  |
| OPA3 | 0.90 | 0.02 | 0.001 |  |  |
| **15. Performance** | | | | 0.50 | 0.90 |
| ITP | 0.52 | 0.05 | 0.001 |  |  |
| ITA | 0.72 | 0.04 | 0.001 |  |  |
| ITPA | 0.73 | 0.03 | 0.001 |  |  |
| TP | 0.53 | 0.05 | 0.001 |  |  |
| TA | 0.83 | 0.04 | 0.001 |  |  |
| TPA | 0.84 | 0.02 | 0.001 |  |  |
| OP | 0.61 | 0.05 | 0.001 |  |  |
| OA | 0.76 | 0.03 | 0.001 |  |  |
| OPA | 0.76 | 0.03 | 0.001 |  |  |

Note: S.E. = standard errors; AVE = average variance extracted; C.R. = composite reliability; MAS = managerial autonomy support; CAS = collegial autonomy support; ACT = autonomy crafting; ITP = individual task proficiency; ITA = individual task adaptivity; ITPA = individual task proactivity; TP = team member proficiency; TA = team member adaptivity; TPA = team member proactivity; OP = organisational member proficiency; OA = organisational member adaptivity; OPA = organisational member proactivity.

**Table S2**

*Correlation and Reliability Coefficients*

|  | MAS | CAS | ACT | SATISFY | ENGAGE | ITP | ITA | ITPA | TP | TA | TPA | OP | OA | OPA | PERFORM |
| --- | --- | --- | --- | --- | --- | --- | --- | --- | --- | --- | --- | --- | --- | --- | --- |
| MAS | (0.94) |  |  |  |  |  |  |  |  |  |  |  |  |  |  |
| CAS | 0.22 | (0.95) |  |  |  |  |  |  |  |  |  |  |  |  |  |
| ACT | 0.32 | 0.14 | (0.65) |  |  |  |  |  |  |  |  |  |  |  |  |
| SATISFY | 0.66 | 0.32 | 0.44 | (0.90) |  |  |  |  |  |  |  |  |  |  |  |
| ENGAGE | 0.42 | 0.20 | 0.26 | 0.69 | (0.83) |  |  |  |  |  |  |  |  |  |  |
| ITP | 0.13 | 0.13 | 0.10 | 0.21 | 0.23 | (0.82) |  |  |  |  |  |  |  |  |  |
| ITA | 0.18 | 0.18 | 0.14 | 0.30 | 0.32 | 0.37 | (0.75) |  |  |  |  |  |  |  |  |
| ITPA | 0.19 | 0.18 | 0.15 | 0.30 | 0.33 | 0.38 | 0.53 | (0.90) |  |  |  |  |  |  |  |
| TP | 0.14 | 0.13 | 0.11 | 0.22 | 0.24 | 0.27 | 0.38 | 0.39 | (0.78) |  |  |  |  |  |  |
| TA | 0.21 | 0.20 | 0.16 | 0.34 | 0.37 | 0.43 | 0.60 | 0.61 | 0.44 | (0.78) |  |  |  |  |  |
| TPA | 0.22 | 0.21 | 0.17 | 0.35 | 0.38 | 0.44 | 0.61 | 0.62 | 0.45 | 0.70 | (0.91) |  |  |  |  |
| OP | 0.16 | 0.15 | 0.12 | 0.25 | 0.27 | 0.31 | 0.44 | 0.45 | 0.32 | 0.50 | 0.51 | (0.84) |  |  |  |
| OA | 0.19 | 0.18 | 0.15 | 0.31 | 0.34 | 0.39 | 0.55 | 0.55 | 0.40 | 0.63 | 0.64 | 0.46 | (0.78) |  |  |
| OPA | 0.19 | 0.18 | 0.15 | 0.31 | 0.34 | 0.39 | 0.55 | 0.56 | 0.40 | 0.63 | 0.64 | 0.46 | 0.57 | (0.89) |  |
| PERFORM | 0.26 | 0.24 | 0.20 | 0.41 | 0.45 | 0.52 | 0.72 | 0.73 | 0.53 | 0.83 | 0.84 | 0.61 | 0.76 | 0.76 | (0.94) |

Notes: MAS = managerial autonomy support; CAS = collegial autonomy support; ACT = autonomy crafting; SATISFY = autonomy satisfaction; ENGAGE = engagement; ITP = individual task proficiency; ITA = individual task adaptivity; ITPA = individual task proactivity; TP = team member proficiency; TA = team member adaptivity; TPA = team member proactivity; OP = organisational member proficiency; OA = organisational member adaptivity; OPA = organisational member proactivity; PERFORM = performance; McDonald’s ordinal omegas presented in brackets diagonally.
